# Defining the Alloreactive T Cell Repertoire Using High-Throughput Sequencing of Mixed Lymphocyte Reaction Culture

**DOI:** 10.1371/journal.pone.0111943

**Published:** 2014-11-03

**Authors:** Ryan O. Emerson, James M. Mathew, Iwona M. Konieczna, Harlan S. Robins, Joseph R. Leventhal

**Affiliations:** 1 Adaptive Biotechnologies Corporation, Seattle, Washington, United States of America; 2 Department of Surgery, Comprehensive Transplant center, Northwestern University, Chicago, Illinois, United States of America; 3 Department of Microbiology-Immunology, Northwestern University, Chicago, Illinois, United States of America; 4 Public Health Sciences Division, Fred Hutchinson Cancer Research Center, Seattle, Washington, United States of America; University of Nebraska-Lincoln, United States of America

## Abstract

The cellular immune response is the most important mediator of allograft rejection and is a major barrier to transplant tolerance. Delineation of the depth and breadth of the alloreactive T cell repertoire and subsequent application of the technology to the clinic may improve patient outcomes. As a first step toward this, we have used MLR and high-throughput sequencing to characterize the alloreactive T cell repertoire in healthy adults at baseline and 3 months later. Our results demonstrate that thousands of T cell clones proliferate in MLR, and that the alloreactive repertoire is dominated by relatively high-abundance T cell clones. This clonal make up is consistently reproducible across replicates and across a span of three months. These results indicate that our technology is sensitive and that the alloreactive TCR repertoire is broad and stable over time. We anticipate that application of this approach to track donor-reactive clones may positively impact clinical management of transplant patients.

## Introduction

Cellular immune response is the most important mediator of transplant rejection and a major barrier to transplant tolerance [1]–[3]. It is largely mediated by memory T cell populations specific for allo-peptides presented either on allo-MHC (direct antigen presentation) or on self-MHC (indirect antigen presentation) [3]–[5]. Positive selection in the thymus requiring immature T cells to have some binding affinity for self-HLA means that a significant proportion of mature T cells also have off-target specificity for allo-HLA alleles. Negative selection removes T cells specific for self-peptides presented on self-HLA, buts leaves T cells specific for self-peptides presented on allo-HLA [6]–[12]. The production of the alloreactive T cell repertoire is further complicated by molecular mimicry. Thus, in one well-studied example a public T cell response specific to EBV in the context of HLA-B*08:01 has been shown to exhibit cross-reactivity with a self-peptide presented by HLA-B*44:02 [13]–[16]. These cross-reactive T cells have been observed in HLA-B*08:01/HLA-B*44:02 mismatched lung allografts, suggesting direct clinical relevance for this mode of T cell alloreactivity [17]. Even in individuals with no history of allo-HLA sensitization, viral exposure or vaccine administration can create HLA cross-reactive memory T cells [18]–[22].

Many studies have identified public and private alloreactive T cell clones that can be primed by a variety of immunogenic events. However, while public T cell clones may play an important role in specific exposures they represent a very small proportion of the entire T cell repertoire; investigating private T cell specificities allows for a much broader view of the alloreactive T cell repertoire but private T cell responses must be measured anew in each subject.

It is our hypothesis that the alloreactive T cell repertoire can be studied by performing mixed lymphocyte reaction cultures [23], [24], followed by molecular analysis of clonotypes thus generated. The availability of high-throughput sequencing of rearranged T cell receptor genes, which act as unique molecular tags for each clonal population, now allows for unprecedented depth and accuracy in the characterization of T cell repertoires. Here, we employ this high-throughput TCR sequencing to test our hypothesis by thoroughly interrogating the alloreactive T cell repertoire between three pairs of healthy adult subjects as well as the persistence of alloreactive T cell clones across biological replicates and across time.

## Methods

### Subjects

Human peripheral blood samples were obtained from laboratory volunteers under a protocol following written informed consent approved and supervised by a Northwestern University Institutional Review Board. These healthy volunteers were HLA-typed by the Northwestern HLA laboratory using molecular methods (reverse sequence specific oligonucleotide probe hybridization).

### Mixed Lymphocyte Reaction (MLR) Culture and Alloreactive Responding Cell Isolation

Peripheral blood mononuclear cells (PBMC) were isolated using Ficoll-Hypaque. The responder cells were labeled with CFSE and the stimulator cells labeled with PKH26 as described previously [25], [26]. The responders and stimulators were matched for 1 HLA- DR antigen to mimic the minimum requirement for some clinical transplants [27]. The PKH26 labeled stimulator cells were also irradiated at 3000 rads. The responder and stimulator cells were cultured in bulk in 15% normal AB serum containing RPMI 1640 culture medium (NAB-CM) at 1×10^6^/ml each. After 7 days these were harvested and the proliferating responders were then sorted on FACSAria (BD, San Jose, CA) by gating on the CFSE dim or negative cells after gating out both CFSE high non-proliferating and the very few PKH26^+^ stimulator cells that still survived.

In parallel, flow cytometric analysis of the above MLR cultures was performed to determine which subsets of responder cells proliferated in response to allostimulation, using fluorochrome conjugated monoclonal antibodies. The data were acquired on an FC500 flow cytometer (Beckman-Coulter) and analyzed for cell subsets by gating on the CFSE dim or negative cells after gating out both CFSE high non-proliferating and the very few PKH26^+^ stimulator cells [25], [26]. Additionally, standard 7-day ^3^H-thymidine incorporation assays were also performed to monitor the strength of the MLR responses as described previously [25], [26].

### High-Throughput TCRβ Sequencing

Genomic DNA was extracted from cell samples using Qiagen DNeasy Blood extraction Kit (Qiagen, Gaithersburg, MD, USA). We sequenced the CDR3 region of rearranged TCRβ genes; the TCRβ CDR3 region was defined according to the IMGT collaboration [28]. TCRβ CDR3 regions were amplified and sequenced using previously-described protocols [29], [30]. Briefly, a multiplexed PCR method was employed using a mixture of 60 forward primers specific to TCR Vβ gene segments and 13 reverse primers specific to TCR Jβ gene segments. Reads of 87 bp were obtained using the Illumina HiSeq System. Raw HiSeq sequence data were preprocessed to remove errors in the primary sequence of each read, and to compress the data. A nearest neighbor algorithm was used to collapse the data into unique sequences by merging closely related sequences, to remove both PCR and sequencing errors.

### PCR Template Abundance Estimation

To estimate the average read coverage per input template in our PCR and sequencing approach, we employed a set of approximately 850 unique types of synthetic TCR analog, comprising each combination of Vβ and Jβ gene segments [31]. These molecules were included in each PCR reaction at very low concentration so that only some types of synthetic template were observed. Using the known concentration of the synthetic template pool, we simulated the relationship between the number of observed unique synthetic molecules and the total number of synthetic molecules added to the reaction (this is very nearly one-to-one at the low concentrations we employed). These molecules then allowed us to calculate for each PCR reaction the mean number of sequencing reads obtained per molecule of PCR template, and thus to estimate the number of T cells in the input material bearing each unique TCR rearrangement.

## Results and Discussion

### Isolation of the Alloreactive T Cell Repertoire

**Figure 1:**
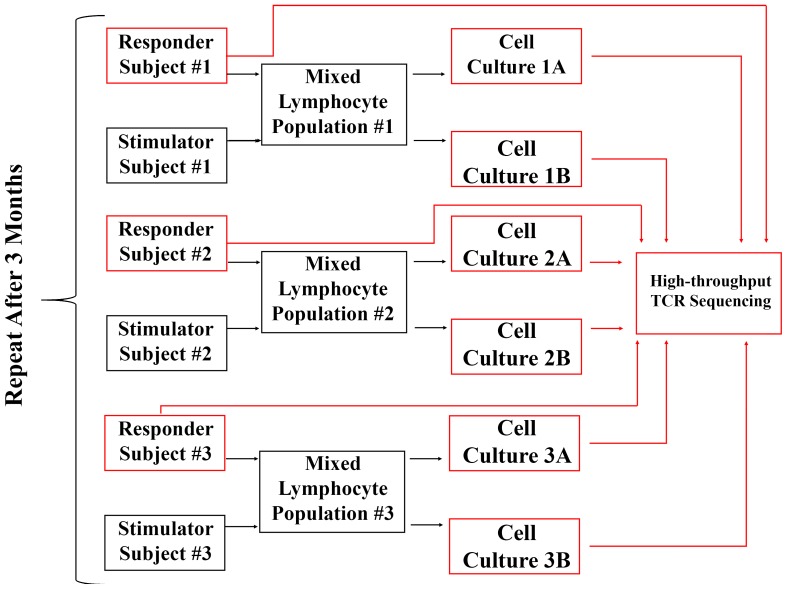
Experimental design. We assayed three pairs of healthy adult subjects using mixed lymphocyte reaction cultures. For each pair, lymphocytes from a responder subject were mixed with inactivated lymphocytes from a stimulator subject and cultured in duplicate. Uncultured freshly isolated PBMC from the responder as well as proliferating T cell populations from the duplicate cultures were subjected to high-throughput sequencing: we sequenced nine samples in total across the three pairs of subjects. Three months later, the experiments were repeated to generate nine more samples for high-throughput TCRβ sequencing.

In order to study the breadth, clonal structure and dynamics of the alloreactive T cell repertoire, we performed a one-way mixed lymphocyte culture using CFSE-labeled responder cells and PKH26-labeled stimulator cells on each of three pairs of healthy adult subjects [25], [26], with cell culture performed in duplicate. Three months after the first experiment, we repeated this cell culture protocol for the same three pairs of subjects. In total, we generated 18 samples of T cells, comprising six samples from each pair of subjects: uncultured total PBMC and purified proliferating T cells from duplicate MLR, at baseline and after three months (Figure 1 summarizes experimental design).

**Figure 2:**
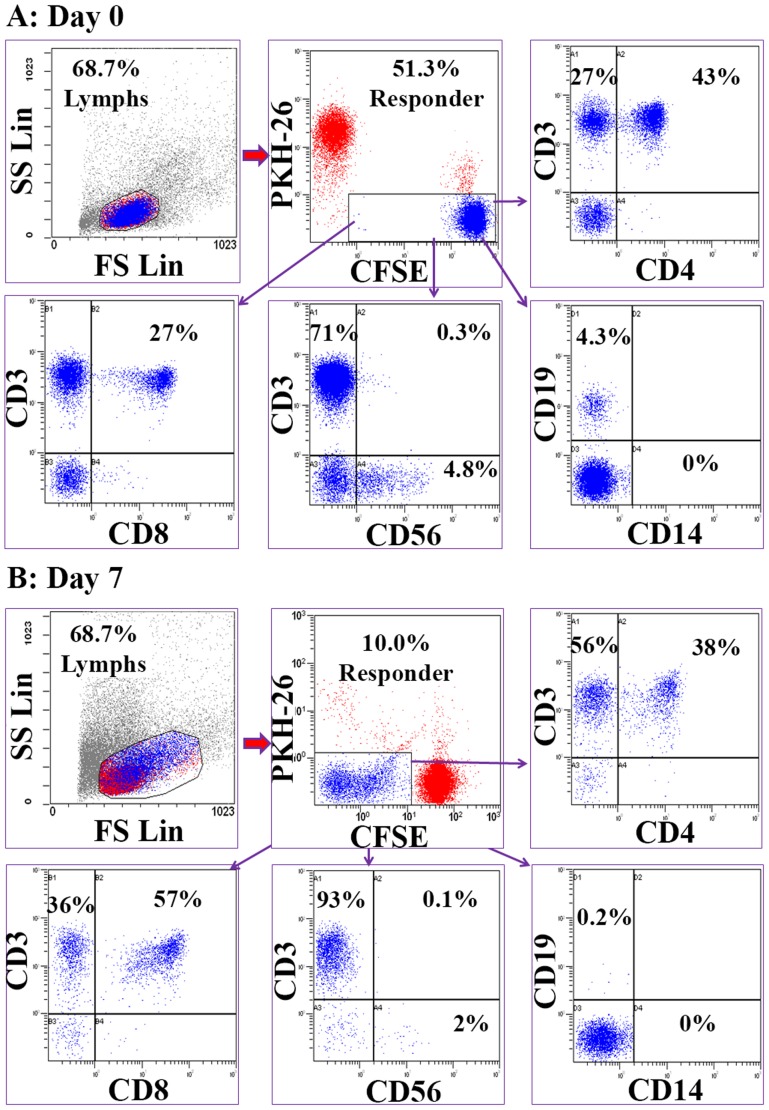
Alloreactive Cellular Subset Profile Generated in MLR. Bulk MLRs were prepared as described in Materials and Methods. The cellular makeup of responder cell populations were delineated at the onset and after 7 days in culture using fluorochrome coupled monoclonal antibodies. The cells were analyzed first by gating on lymphocytes and then after gating either on total CFSE positive responder cells (A: Day 0) or on CFSE diluted proliferating responder cells (B: Day 7).

For each MLR reaction, after 7 days the proliferating responders were sorted by gating on the CFSE dim or negative cells after gating out both CFSE high non-proliferating and the very few PKH26^+^ stimulator cells that still survived (Figure 2A). The proliferating cells consisted of 40.3±4.7% CD3^+^CD4^+^ and 57.2±5.1% CD3^+^CD8^+^ T cells as well as minor subset of CD56^+^ NK cells (Figure 2B). Each population of uncultured PBMC or proliferating T cells was subjected to amplification and high-throughput sequencing of the CDR3 region of TCRβ, which somatically rearranges during T cell maturation and acts as a unique molecular tag for each clonal population of T cells. Sequencing results are presented in Table 1.

**Table 1:** Summary of TCRβ sequencing results.

*Sample*	*T cells assayed (estimated)* a	*Unique TCRβ sequences*	*Sequencing reads* b
*Fresh PBMC sample #1, 0 months*	4,336,812	750,211	51,160,577
*Fresh PBMC sample #2, 0 months*	4,774,312	1,375,340	46,370,325
*Fresh PBMC sample #3, 0 months*	4,016,260	991,848	33,633,101
*Fresh PBMC sample #1, 3 months*	713,990	264,159	17,437,692
*Fresh PBMC sample #2, 3 months*	1,847,987	1,046,492	23,507,950
*Fresh PBMC sample #3, 3 months*	2,197,064	1,061,154	18,766,880
*Proliferated MLR responder #1A, 0 months*	1,885,973	33,677	23,366,016
*Proliferated MLR responder #1B, 0 months*	1,997,723	33,387	26,098,554
*Proliferated MLR responder #2A, 0 months*	1,575,201	79,174	24,704,053
*Proliferated MLR responder #2B, 0 months*	1,527,643	68,505	13,832,785
*Proliferated MLR responder #3A, 0 months*	3,372,150	58,382	37,022,643
*Proliferated MLR responder #3B, 0 months*	3,190,902	53,316	23,126,368
*Proliferated MLR responder #1A, 3 months*	640,366	57,778	12,741,642
*Proliferated MLR responder #1B, 3 months*	587,681	53,260	9,806,707
*Proliferated MLR responder #2A, 3 months*	1,022,417	68,565	10,736,335
*Proliferated MLR responder #2B, 3 months*	522,273	53,337	10,679,864
*Proliferated MLR responder #3A, 3 months*	685,126	64,615	9,788,942
*Proliferated MLR responder #3B, 3 months*	760,990	67,586	10,999,866
	**35,654,870**	**6,180,786**	**403,780,300**

a see Methods.

b the total number of 87-bp sequencing reads generated.

### Size of the Alloreactive T Cell Repertoire

To determine the number of T cell clonal lineages involved in the alloreactive T cell response, we analyzed the number of unique CDR3 sequences observed in the proliferated T cell samples in comparison to uncultured bulk T cells from the same subjects. We defined alloreactive T cell clones as those observed in at least 10 cells in the proliferated sample and unobserved in the uncultured T cell sample, or T cells whose frequency in the proliferated sample was at least ten-fold higher than in the uncultured T cell sample. We defined two sets of alloreactive T cell clones: low-abundance alloreactive clones (below the threshold of detection in the subject's baseline T cell repertoire) and high-abundance alloreactive clones (present at measurable frequency in the subject's baseline T cell repertoire). On average, we observed 14,000 alloreactive T cell clones in each experiment; 84% of alloreactive T cell clones were low-abundance before proliferation, but in total low-abundance clones made up 40% and high-abundance clones made up 60% of the alloreactive T cell repertoire when weighting by post-proliferation clonal abundance (Table 2). While the number of proliferated low-abundance clones varied considerably, variation in the number of high-abundance (thus, presumably antigen-experienced) T cell clones between subjects was much smaller, at about 2,000 clones in each of the six experiments. These data indicate that thousands of different clonal populations of T cells comprise the alloreactive T cell repertoire.

**Table 2:** Size of the alloreactive T cell repertoire.

	*Mean (N = 6)*	*SD*	*% of proliferated T cells*
***Number of alloreactive clones***	13750	6823	100%
***Low-abundance pre-culture*** a	11610	6494	40.0%
***High-abundance pre-culture*** b	2140	539	60.0%

a unobserved in pre-culture sample and ≥10 T cells after MLR.

b present in pre-culture sample and ≥10× enriched after MLR.

### Reproducibility of the Alloreactive T Cell Repertoire

**Figure 3:**
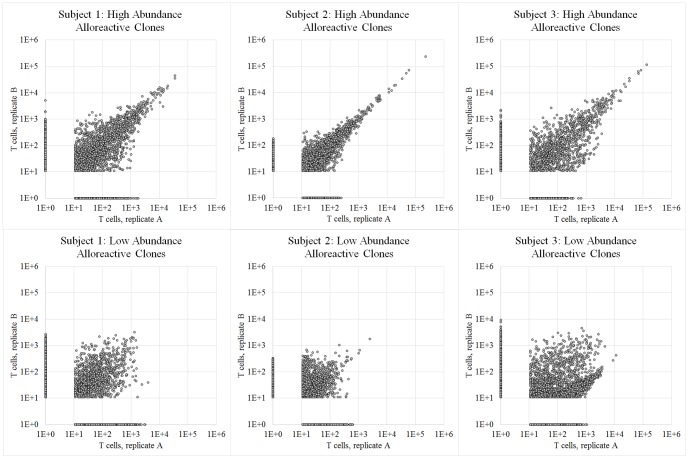
T cell clonal frequency among biological replicates of mixed lymphocyte culture. Above are six scatter plots showing the number of T cells bearing each unique CDR3 sequence in replicate mixed lymphocyte culture experiments performed on three pairs of healthy adult subjects. Each column corresponds to one pair of subjects; the top row of plots show T cell clones that were previously observed in a pre-MLR sample of peripheral T cells (high-abundance), and the bottom row of plots show T cell clones unobserved in a pre-MLR sample of peripheral T cells (low-abundance). Each point represents a unique T cell clone, and points are plotted at (# of observed T cells +1), so that clones unobserved in one sample are plotted on the axes.

To assay the consistency of the alloreactive T cell repertoire, we examined the persistence of each T cell clone. After defining high-abundance and low-abundance alloreactive T cells, we compared the set of alloreactive T cell clones generated in duplicate cell culture experiments (Figure 3). In each subject, essentially all clones that were highly expanded in proliferated cell culture assorted to the high-abundance subset (i.e., were present at appreciable frequency in the peripheral T cell repertoire to begin with). Reproducibility between duplicate cell culture experiments was high among this set of abundant and highly alloreactive T cell clones (average r^2^ among three subjects = 0.96), indicating that when presented with identical stimuli these clonal populations of T cells responded in a very reproducible manner.

**Figure 4:**
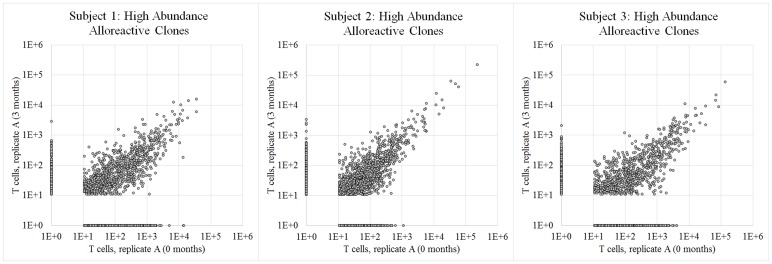
T cell clonal frequency among temporal replicates of mixed lymphocyte culture. Above are three scatter plots showing the number of T cells bearing each unique CDR3 sequence in replicate mixed lymphocyte culture experiments performed three months apart on each of three pairs of healthy adult subjects. Considering only T cell clones previously observed in a pre-MLR T cell sample from each time-point and enriched at least ten-fold after mixed lymphocyte culture, each point represents a unique T cell clone and points are plotted at (# of observed T cells +1) so that clones unobserved in one sample are plotted on the axes.

Since our replicate cell culture experiments did not address the stability of the alloreactive T cell repertoire over time, we repeated the T cell isolation and duplicate MLR experiments with the same three pairs of subjects three months after our initial experiment. Specifically, we hypothesized that high-abundance alloreactive clones, which we presume to represent memory T cells due to their frequency in the peripheral T cell repertoire, should be stable over time and thus should remain in the alloreactive T cell compartment. Figure 4 presents the high-abundance T cell repertoire after three months in each pair of subjects. Many T cell clones identified as part of the high-abundance alloreactive T cell repertoire at baseline were observed in the high-abundance alloreactive T cell repertoire three months later, at similar clonal frequencies (Figure 4; average r^2^ = 0.78). To quantify similarity between sets of T cells, we calculated a TCR overlap metric (the proportion of T cells belonging to clones found in both samples) [29]. Table 3 presents the TCR overlap between duplicate cell culture experiments and between experiments spaced three months apart. While duplicate cell culture experiments generated more concordant sets of alloreactive T cell clones than experiments from different time-points, overlap between different time-points was nonetheless quite high (mean overlap = 0.97 for duplicate experiments vs. 0.87 across time-points). We hypothesize that the lower overlap over time might be due to the emergence of naïve T cell clones of exceptional size which would not be expected to persist in the periphery and/or the noise in the estimation of absolute cellular abundance could have caused a subset of low-abundance clones to be erroneously classified as high-abundance in our experiment [32]–[34].

**Table 3:** TCR overlap between biological & temporal replicate mixed lymphocyte culture experiments.

	*Biological replicates (N = 2)* a	*Temporal replicates (N = 4)* b
***High-abundance pre-culture*** c		
*Subject 1*	0.96	0.78
*Subject 2*	0.98	0.93
*Subject 3*	0.98	0.89
*Average*	**0.97**	**0.87**
***Low-abundance pre-culture*** d		
*Subject 1*	0.54	0.15
*Subject 2*	0.43	0.06
*Subject 3*	0.67	0.08
*Average*	**0.55**	**0.10**

a MLR Cultured in duplicate.

b MLR performed at three months apart.

c Present in pre-culture sample and ≥10× enriched after MLR.

d Unobserved in pre-culture sample and ≥10 T cells after MLR.

The low-abundance alloreactive T cell clones, however, showed lower reproducibility between duplicate cell culture experiments (Table 3, bottom) and appeared to be considerably more transient. Comparisons between biological duplicates were much more concordant than comparisons between time-points (mean overlap = 0.55 for duplicate experiments vs. 0.10 across time-points). Several hypotheses may explain why T cell clones were not reproducibly found in the low-abundant alloreactive T cell compartment; first, the lower overlap between biological replicates is mostly due to sample error (most unique T cell lineages are at very low abundance, and we cannot reliably find a T cell clone in two biological replicates unless it is present in at least several cells); second, the even lower reproducibility after three months can be attributed to a preponderance of newly emerged naïve T cell clones among this subset; lastly, these clones may represent memory T cell populations that did not persist at detectable levels in the periphery over the intervening time [32]–[34].

Taken together, our TCR repertoire analysis is highly sensitive and reproducible. Further, our results indicated that a majority of the alloreactivity observed between three pairs of healthy adults was attributable to a set of several thousand T cell clones, present at reasonably high frequency in the peripheral T cell repertoire, whose alloreactive potential remained stable over at least several months. While we cannot conclusively demonstrate using TCR sequencing that the proliferating clones identified are specifically alloreactive, our screening algorithm (requiring a T cell clone to represent a 10× higher proportion of the proliferated than the fresh sample) should ensure that only a minimal number of nonspecifically-proliferating clones are identified. Likewise, we do not anticipate that our method will reliably identify all T cell clones which will react to and infiltrate an allograft. However, we expect neither tracking of some irrelevant T cell clones nor failure to track some genuinely alloreactive clones should compromise clinical utility so long as a sufficient number of truly alloreactive clones are also identified and tracked, providing a means of quantitating the host cellular immune response to the allograft.

We anticipate that application of our approach to transplantation could have a positive impact in the clinical management of patients. This is to be achieved by performing donor-specific MLR at transplant to pre-define the donor-reactive T cell repertoire, and then tracking their presence, abundance and dynamics in recipient primary tissues (e.g. peripheral blood, allograft biopsies, urine) during the post-transplant period. Such an approach will make this technology utilizable in both living donor and deceased donor transplants. We speculate that foreknowledge of the alloreactive T cell repertoire could thus be combined with post-transplant immune profiling in the recipient peripheral blood for non-invasive monitoring of cellular allograft rejection. Conversely, absence of the donor reactive clones from the post-transplant repertoire would indicate immune tolerance.
